# Altered dynamic effective connectivity of the default mode network in type 2 diabetes

**DOI:** 10.3389/fneur.2023.1324988

**Published:** 2024-01-15

**Authors:** Kun Xu, Jun Wang, Guangyao Liu, Jiahao Yan, Miao Chang, Linzhen Jiang, Jing Zhang

**Affiliations:** ^1^Second Clinical School, Lanzhou University, Lanzhou, China; ^2^Department of Magnetic Resonance, Lanzhou University Second Hospital, Lanzhou, China; ^3^Gansu Province Clinical Research Center for Functional and Molecular Imaging, Lanzhou University Second Hospital, Lanzhou, China

**Keywords:** type 2 diabetes mellitus, dynamic effective connectivity, default mode network, independent components analysis, multivariate granger causality analysis

## Abstract

**Introduction:**

Altered functional connectivity of resting-state functional magnetic resonance imaging (rs-fMRI) within default mode network (DMN) regions has been verified to be closely associated with cognitive decline in patients with Type 2 diabetes mellitus (T2DM), but most studies neglected the fluctuations of brain activities—the dynamic effective connectivity (DEC) within DMN of T2DM is still unknown.

**Methods:**

For the current investigation, 40 healthy controls (HC) and 36 T2DM patients have been recruited as participants. To examine the variation of DEC between T2DM and HC, we utilized the methodologies of independent components analysis (ICA) and multivariate granger causality analysis (mGCA).

**Results:**

We found altered DEC within DMN only show decrease in state 1. In addition, the causal information flow of diabetic patients major affected areas which are closely associated with food craving and metabolic regulation, and T2DM patients stayed longer in low activity level and exhibited decreased transition rate between states. Moreover, these changes related negatively with the MoCA scores and positively with HbA1C level.

**Conclusion:**

Our study may offer a fresh perspective on brain dynamic activities to understand the mechanisms underlying T2DM-related cognitive deficits.

## 1 Introduction

Type 2 diabetes mellitus (T2DM) is the most prevalent chronic metabolic disease in the world, with the main trait of glucose metabolism dysregulation (1). Under the effect of long-term hyperglycemia and blood glucose fluctuations, diabetes can cause multiple systemic macrovascular and microvascular lesions and eventually may lead to severe comorbidities in multiple organs (2, 3). Findings from lots of epidemiological studies suggested that people with diabetes have a higher risk of developing dementia and demonstrated inferior performance compared to non-diabetes in a variety of neural and cognitive processes, primarily including information-processing speed, memory, attention, executive function, verbal fluency and so on (4). However, the neuropathophysiological mechanisms underpinning cognitive impairment caused by T2DM are not fully understood.

As indicated by the results of a meta-analysis, the increase and decrease of functional connectivity in T2DM patients was mostly concentrated within and between the default mode network, and these DMN-centered impairments are bound up with cognitive decline (5). One of the more developed parts of the brain, the DMN has the highest functional connectivity, making it a potential candidate for early cognitive impairment in T2DM patients. The main components of DMN involved the precuneus/posterior cingulate cortex (PCC), medial prefrontal cortex (MPFC), lateral temporal cortex (LTC), inferior parietal lobule (IPL) and hippocampus, and this network mainly undertake the functions of self-referencing, emotional processing, memory, spontaneous cognition, and awareness (6). Therefore, exploring the early functional alterations of DMN may offer potential imaging markers on cognitive changes in T2DM patients. Previous study has indicated that cognitive function was associated with lower connectivity within the DMN in patients with T2DM (7). And functional connectivity between the anterior and posterior areas of the DMN and the local efficiency of the DMN were significantly decreased in T2DM, which was closely related to memory and executive functions, the early manifestation of cognitive impairment in T2DM (8–10).

In normal living and during sleeping, studies have demonstrated that the FC within DMN areas is continually changing, and different network states can be seen even within a minute (11–15). However, the majority of the studies discussed above only examined the static functional connectivity within the DMN, and only a few concentrated on dynamic and effective functional connectivity, which can track changes in the strength of the connections between various regions of interest over time and capture spontaneously recurring patterns of functional connectivity (16). According to the available results of casual analysis, patients with T2DM showed altered DEC between the left fusiform gyrus and bilateral lingual gyrus and right medial frontal gyrus (MFG), the right SFG and bilateral frontal regions, as well as between left hippocampus (LHIP) and right hippocampus (RHIP), occipital cortex and cerebellum (17, 18). Effective connectivity network is a graph model consisting of nodes and directed edges, where the nodes represent brain regions and the directed edges portray the causal effect of neural activity exerted by one brain region on another brain region, and the edge-related connection parameter indicates the connection strength of the edge (19, 20). mGCA is one of the methods which used a factor model to downscale the high-dimensional fMRI data, then a multivariate autoregressive model was built in the low-dimensional subspace, and a biased directed coherence algorithm was used to identify the granger causality between brain regions, finally, the identification results were mapped to the high-dimensional state space to obtain the brain effect connectivity network (21). The research methodology has been used in disorders including Alzheimer's, moderate cognitive impairment (MCI), mental illness, and others, and it was verified that the alterations of effective connections were strongly related to the cognitive states of our brain (22–24).

However, we still know so little about how DEC changes within DMN for T2DM patients. According to our hypothesis, abnormal DEC within DMN is related to T2DM's cognitive impairment and is caused by glucose imbalances. In order to identify neuroimaging markers for cognitive deficits in T2DM and how disturbance of glucose metabolism affects this relationship, we investigated DEC differences between T2DM and HCs using mGCA.

## 2 Methods

### 2.1 Participants

A total of 76 participants were enrolled in this study, which consisted of 36 diabetics and 40 healthy controls. T2DM diagnosis adhered to WHO criteria, defined by either a random plasma glucose level ≥11.1 mmol/L with typical diabetes symptoms or a fasting plasma glucose ≥7.0 mmol/L, or an oral glucose tolerance test (OGTT) ≥11.1 mmol/L, the OGTT performing in non-pregnant adults by using a 75-g oral glucose load with examination of the 2-h plasma glucose. Patients were excluded if they had any of the following characteristics: (1) with magnetic resonance contraindications, (2) had or with neurological or psychiatric illness, (3) with macrovascular or microvascular complications (e.g., retinopathy, nephropathy, and neuropathy), and (4) with head injury, alcoholism and mass lesions (e.g., cancer, anemia, and thyroid dysfunction).

### 2.2 Clinical data and neuropsychological test information

The clinical and demographic data of all subjects were gathered. The demographic indices mainly incorporated sex, age, level of education and body mass index (BMI). Blood samples were obtained at 8 A.M., via venipuncture to assess the levels of fasting blood glucose, glycosylated hemoglobin type A1C (HbA1c), and blood lipid content [including cholesterol (CHO), triglycerides (TG), high-density lipoprotein (HDL)]. Additionally, we measured blood pressure [including systolic blood pressure (SBP) and diastolic blood pressure (DBP)] and postprandial blood glucose (PBG). We used the Mini Mental State Examination (MMSE) and the Montreal Cognitive Assessment (MoCA) as two straightforward neuropsychological tests to evaluate the individuals' overall cognitive abilities.

### 2.3 MRI acquisition

MRI data were acquired on a Siemens Verio 3.0T scanner (Siemens, Erlangen, Germany) with 8 head-coil at the Department of Magnetic Resonance of Lanzhou University Second Hospital. During the scan, patients were asked to close their eyes and stay awake. To minimize head movement and isolate noise, earbuds and foam pads were used. Resting-state functional images were obtained employing an axial echo planar imaging (EPI) sequence with the following parameters: repetition time (TR) = 2000 ms; echo time (TE) = 20 ms; flip angle = 90°; slice thickness = 3 mm; in-plane matrix resolution = 64 × 64; field of view (FOV) = 192 × 192 mm^2^; slices = 36; number of total volumes = 180; resolution = 3 × 3 mm^2^; total duration = 6 min. High-resolution 3D T1w images were acquired by employing a magnetization-prepared rapid gradient-echo sequence (MPRAGE) with the following parameters: T*R* = 1900 ms; TE = 2.93 ms; flip angle = 9°; slice thickness = 1 mm; acquisition matrix = 256 × 256; FOV = 256 × 256 mm^2^; resolution = 1 × 1 mm^2^.

### 2.4 Preprocessing

Anatomical images and resting-state fMRI dataset were preprocessed by applying the pipeline fMRIPrep Version 20.2.0 (25). The intensity non-uniformity correction and skull-stripping of T1w images were performed on the platform ANTs. The brain extraction made an auto-coregistration to the OASIS template and acquired the reference, brain-extracted T1w image. Then, spatial normalization of the T1W and standard template (the Montreal Neurosciences Institute 152 Non-linear Asymmetrical template in version 2009c with the resolution of 2 mm^3^) was manipulated through the ANTs' Registration, and meanwhile, generated the brain mask and cost function mask. The brain mask then went through refinement for mediating the ANTs-derived and Free Surfer-derived segmentation. Segmentation of different tissues -cerebrospinal fluid (CSF), white matter (WM) and gray matter (GM) were all segmented using the FSL method.

Preprocessing of the resting-state fMRI data included the following steps: (1) removing the first 10 time points; (2) creating a reference and brain mask; (3) realignment and estimation of head motion; (4) slice-timing correction; (5) susceptibility distortion estimation; (6) alignment to segmented-T1w image; (7) normalization to the standard template (the MNI 152 Non-linear Asymmetrical template in version 2009c); and (8) smoothing with the Gaussian kernel of with the Gaussian kernel of 6 mm full width at half maximum. Every participant's frame had translational or rotational motion parameters < 3 mm and 2°.

### 2.5 Independent component analysis

The Spatial-temporal group ICA was performed in order to the selection of ROIs of DMN by the GIFT software Version 4.0c (University of New Mexico, Albuquerque, NM) implemented in Matlab R2022b, which consist of the following two data-reduction procedures: (1) Firstly, the principal component analysis (PCA), provided an orthonormal space to reduce the group functional images into 150-dimensional subspaces and generate 150 time courses and 100 corresponding spatial maps (ICA components) in accordance with the infomax algorithm which can minimize mutual information to enhance the reliability and validity of outputs; (2) Reconstructing individual-subject spatial maps and associated time courses in reverse is the second step. Twelve ICs were isolated and used as the areas of interest (ROI) of the DMN in the definition of ROIs for the neurocognitive networks under previous research (26). Components were also appraised using the following standards: Peak activation coordinates were largely found in gray matter, there was little spatial overlap with recognized vascular, ventricular, motion, and susceptibility artifacts, and low-frequency fluctuations predominated the time courses.

### 2.6 Dynamic effective connectivity

Then, we investigated DEC between DMN areas in DynamicBC Version 2.1 (27) program using the mGCA module. To obtain the 76 causal impact matrices, the time courses were first divided using the sliding windows method, whose rectangle window size was 18 TRs and step length was 2 TRs. In order to categorize different clusters (corresponding to different states) based on the similarity between matrices and cluster centroids, a k-means clustering method was applied to the windowed EC matrices. And then, we measured the discrepancy of DEC between patients with T2DM and HC in different states using the two-sample *t-*test by using NBS Version 1.2 (FDR; *p* < 0.05).

### 2.7 Causal influence flows analysis and temporal properties analysis

In order to explore the state-directed communications among DM components, we compared the results of two weighted degree measures—the in-weighted degree and the out-weighted degree (28), which are the most widely used parameters of causal influence flows—between T2DMs and controls in each state. The total influence strength from one component to others is the in-weighted degree of that component, and the total influence strength from that component to others is the out-weighted degree. Generally speaking, the component with a high in-weighted degree/out-weighted degree was considered as the hub receptor/generator of the network and was essential to functional integration. We additionally computed the in-out-weighted degree, which is defined as the subtraction of in-weighted degree and out-weighted degree, in order to evaluate net influence for each component. Additionally, we calculated the DEC's temporal properties [including fractional windows (F), mean dwell time (MDT), and number of transitions (NT)] to assess the temporal characteristics of two states (29). To be more precise, MDT was defined as the average number of consecutive windows that belong to each state and indicates the amount of time stay in particular state; F was defined as the percentage of windows that belong to each state; and NT was defined as the transition times between states and implied the stability over time.

### 2.8 Statistical analysis

**Figure 1:**
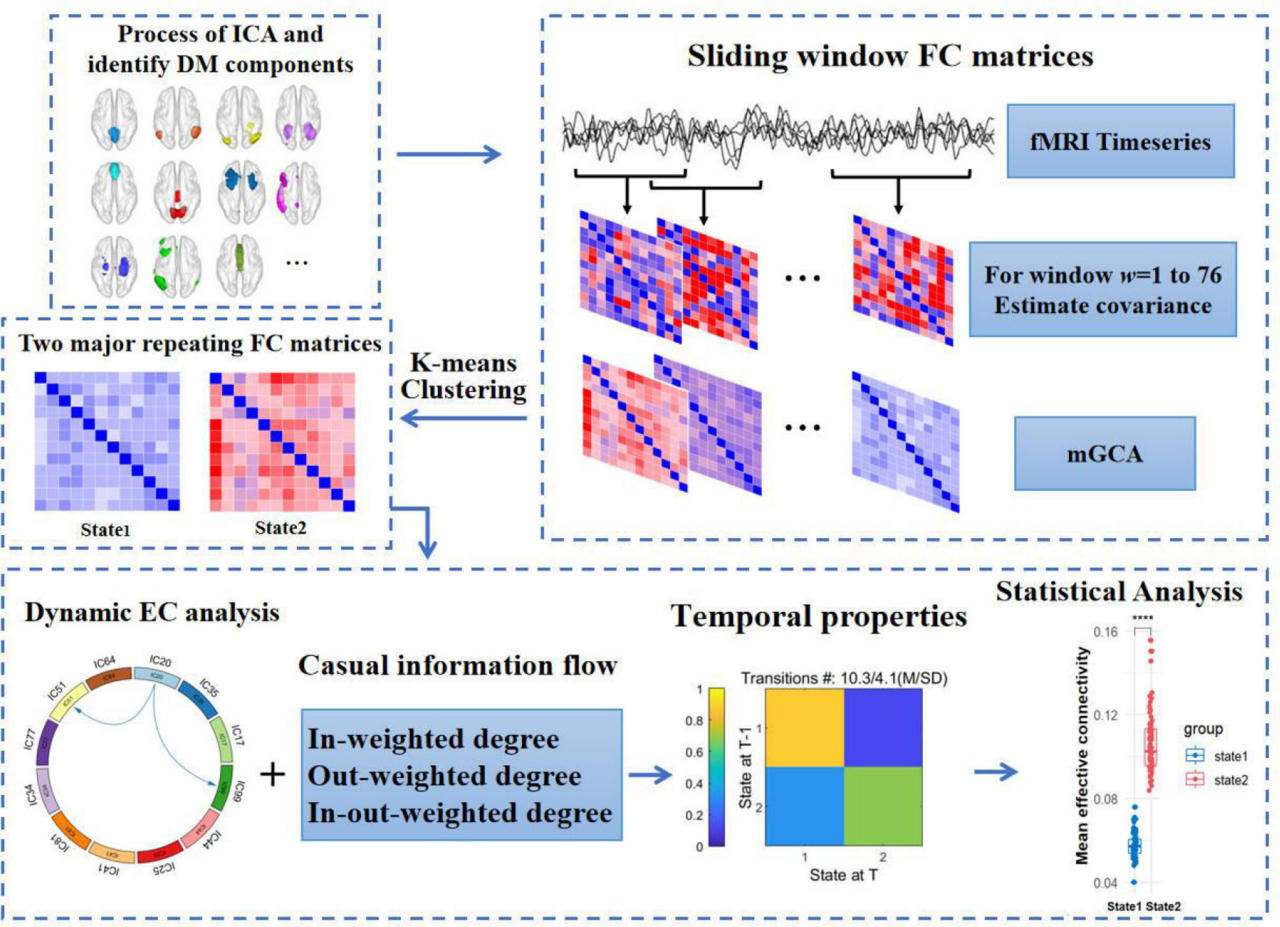
Flow chart of analysis in the study. fMRI, functional magnetic resonance imaging. (1) The DM components acquired from independent components analysis. (2) Sliding windows method with the rectangle window size of 18 TRs and step length of 2 TRs was used to obtain causal impact matrices. (3) K-means clustering categorized all matrices to different clusters. (4) Measuring the difference of dynamic effective connectivity, casual information flow and temporal properties between T2DM and HCs. DM components, default mode components.

The connections between altered DEC parameters and clinical characteristics were examined using Spearman's correlation analysis. False discovery rate (FDR; *p* < 0.05) was used to adjust the results for multiple comparisons after statistical analyses were carried out using SPSS Version 26.0. See Figure 1 for illustration. Besides, in order to investigate the consistency of DEC clusters/states with varied processing sliding window parameters, we reset the rectangle windows of the sliding windows method to 30 TRs and 12 TRs and the step lengths of that to 1 TRs and 3 TRs respectively in Dynamic BC, and calculated their temporal properties. Then, we measured the difference of intraclass correlation coefficient (ICC) between the setups of sliding windows described above and covered in our paper by applying two-way random effects model, and the ICC's single measures of fractional windows were 0.903 and 0.887 respectively (*p* < 0.0001).

## 3 Result

### 3.1 Demographic, clinical, and cognitive characteristics

As shown in Table 1, we can see that the T2DM group consists of 20 males and 16 females, these patients with the average age of 55.94 ± 7.69 years, average years of education of 13.31 ± 2.48 years, average disease duration of 6.56 ± 5.09 years and average body mass index (BMI) of 21.67 ± 1.51 kg/m^2^. The HC group included 19 males and 21 females, and the average age, years of education and BMI were 54.30 ± 6.76 years, 13.60 ± 2.96 years, 21.01 ± 2.73 kg/m^2^ respectively. By the means of two-sample *t-*test, we were unable to identify any statistically significant differences between the two groups in terms of sex, age, education and BMI.

**Table 1:** Demographic, clinical and cognitive characteristics.

**Characteristics**	**T2DM patients**	**Healthy controls**	***P*-value**	***T-*value**
	**(*****n*** = **36)**	**(*****n*** = **40)**		
Age (years)	55.94 ± 7.69	54.30 ± 6.76	0.33	−0.992
Sex (male)	20 (36)	19 (40)	0.37	−0.912
Education (years)	13.31 ± 2.48	13.60 ± 2.96	0.64	0.467
Disease duration (years)	6.56 ± 5.09	—-	< 0.01^**^	−8.157
Body mass index (kg/m^2^)	21.67 ± 1.51	21.01 ± 2.73	0.2	−1.295
Fasting glucose (mmol/L)	10.98 ± 3.56	4.27 ± 0.39	< 0.01^**^	−11.829
2h-postprandial glucose (mmol/L)	15.72 ± 5.74	8.74 ± 0.70	< 0.01^**^	−7.975
HbA1c (%) (mmol/mol)	10.58 ± 3.17	4.92 ± 0.50	< 0.01^**^	−11.151
Total cholesterol (mmol/L)	4.18 ± 0.78	4.03 ± 0.59	0.37	−0.903
Triglycerides (mmol/L)	1.79 ± 0.92	1.40 ± 0.74	0.02^*^	−2.372
High-density lipoprotein (mmol/L)	1.12 ± 0.28	1.16 ± 0.37	0.52	0.644
Systolic blood pressure (mmHg)	117.64 ± 8.72	120.95 ± 9.51	0.12	1.577
Diastolic blood pressure (mmHg)	73.08 ± 12.0	68.35 ± 11.03	0.08	−1.794
MoCA	27.44 ± 1.87	27.83 ± 1.50	0.33	0.982
MMSE	28.78 ± 1.46	29.13 ± 0.94	0.22	1.248

^*^p < 0.05 and ^**^p < 0.01. HbA1c, glycosylated hemoglobin type A1C; MoCA, the Montreal Cognitive Assessment; and MMSE, the Mini Mental State Examination.

Additionally, indicator values such as fasting blood glucose (FBG), 2-h postprandial blood glucose, HbA1c, and triglycerides (TG) were significantly higher in the T2DM group compared to the HC group (*p* < 0.05), but there was no difference in CHO, HDL, blood pressure and cognitive scores between two groups (*p* > 0.05), details shown in Table 1.

### 3.2 ICA results

**Figure 2:**
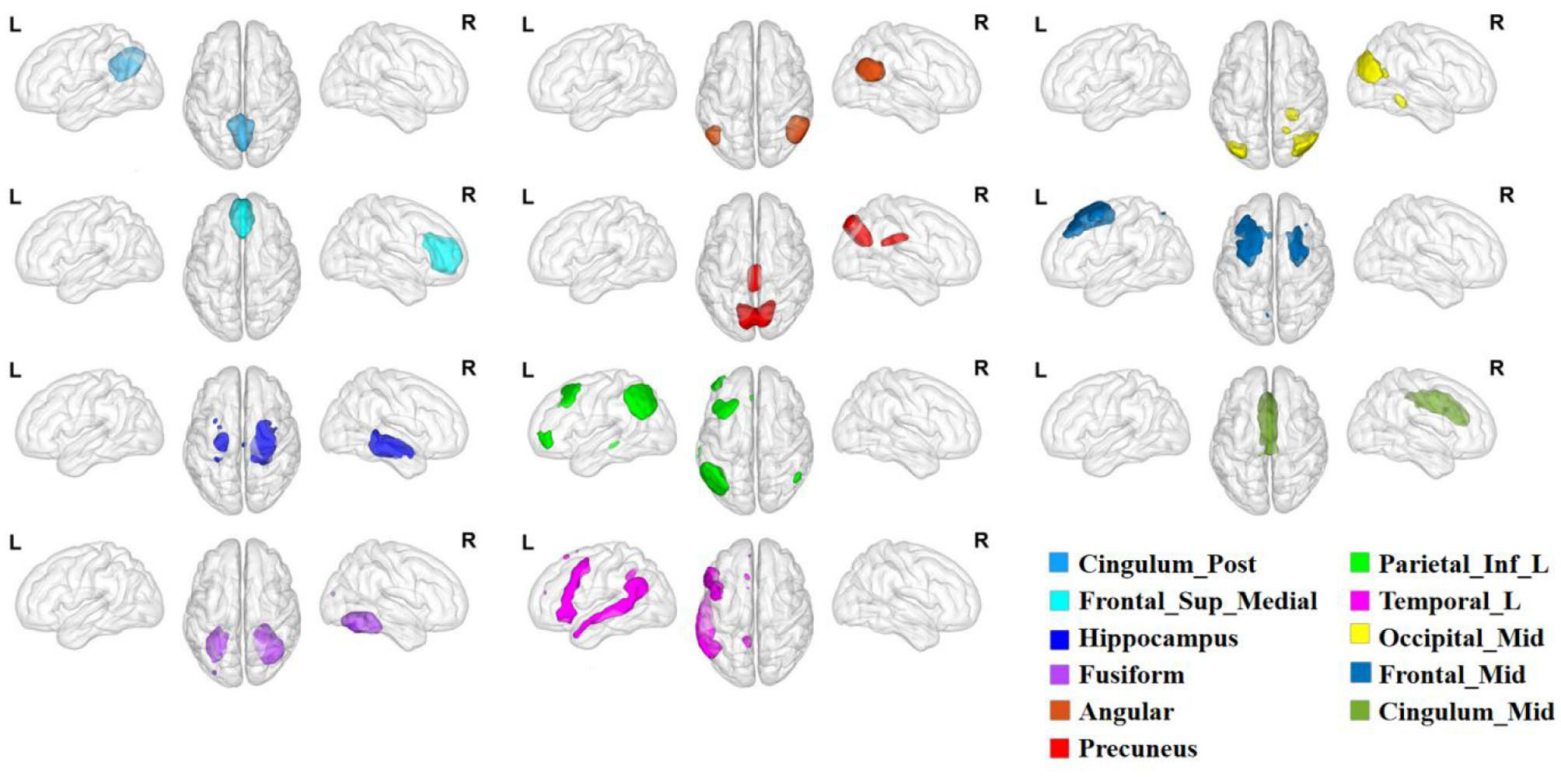
The distribution of DM components. DM components, default mode components.

Through the ICA, we obtained 12 components in the DMN, including the bilateral posterior cingulate cortex (PCC; IC17), medial prefrontal cortex (mPFC; IC20), hippocampus (Hip; IC25), fusiform gyrus (FFG; IC34), angular gyrus (Ang; IC35, IC44), precuneus (PCU; IC41), middle occipital gyrus (MOG; IC77), middle frontal gyrus (MFG; IC81), middle cingulate cortex (MCC; IC99), and the left inferior parietal lobule (IPL; IC51) and temporal lobe (TL; IC64). The detailed information of these regions are shown in Figure 2 and Table 2.

**Table 2:** Detailed information of DM components.

**Independent component**	**Anatomical regions**	**Cluster size**	**MNI coordinates**			***T*-value**
			**X**	**Y**	**Z**	
IC17	Cingulum_Post_L&_R	331	0	−54	30	36.2187
IC20	Frontal_Sup_Medial_L&_R	1,015	0	48	12	26.4431
IC25	Hippocampus_L&_R	833	4	−34	−32	22.9179
IC34	Fusiform_L&_R	1,910	10	−100	−26	22.8899
IC35	Angular_L	736	−6	−70	32	29.1449
IC41	Precuneus_L&_R	1,897	0	−54	30	26.5415
IC44	Angular_R	1,153	46	−66	42	30.1579
IC51	Parietal_Inf_L	1,810	54	−54	20	29.1449
IC64	Temporal_L	2,155	−50	−60	22	20.6183
IC77	Occipital_Mid_L&_R	1,360	4	−160	62	30.9279
IC81	Frontal_Mid_L&_R	1,793	0	26	116	20.7429
IC99	Cingulum_Mid_L&_R	1,726	−4	22	30	22.2497

DM components, default mode components.

### 3.3 DEC results

**Figure 3:**
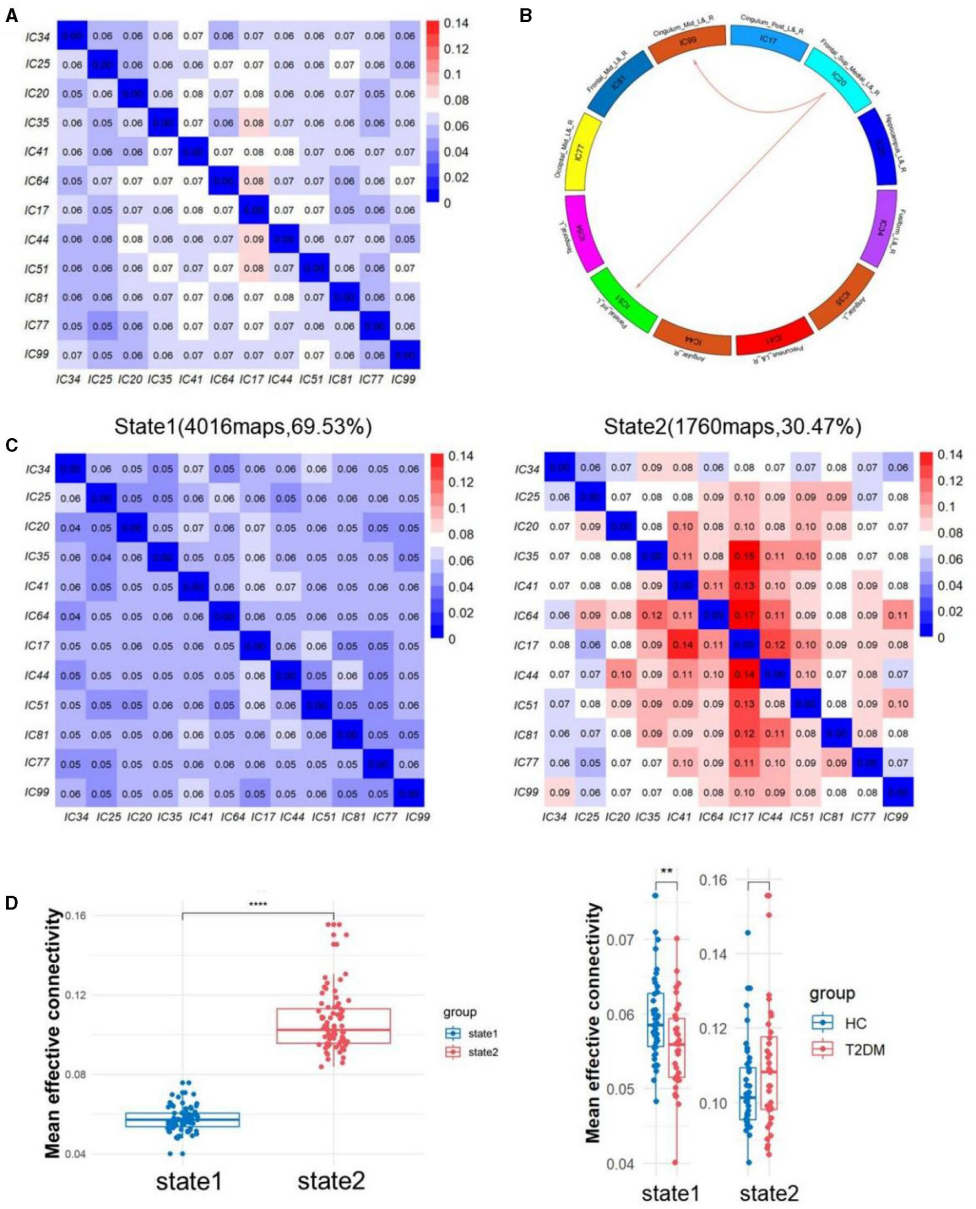
**(A)** The averaged static effective connectivity (EC) between DM component pairs was computed using an entire scan. **(B)** Decreased dynamic EC from IC20 to IC51 and IC99. **(C)** Centroid matrices for two states and the occurrences and the percentage of the two states. **(D)** Differences of mean effective connectivity between two states and between two groups in each state. **p < 0.01 and ****p < 0.0001.

The sliding window approach was used to collect a total of 5,776 EC matrices from 76 participants. K-means clustering analysis then separated all of the matrices into two states, as seen in Figure 3. State 1 occupied 69.53% of the matrices, whereas state 2 took up 30.46% of them all. Compared to State 1, State 2 exhibited significantly more active inter-regional communication. Notably, State 1 demonstrated pronounced differences in DEC between T2DM patients and healthy individuals. We discovered that T2DM had lower EC from IC20 to IC51 and IC99 in state1 compared to non-diabetic controls by using NBS. In state 2, there was no discernible difference between the two groups.

### 3.4 Causal information flows and temporal properties

**Figure 4:**
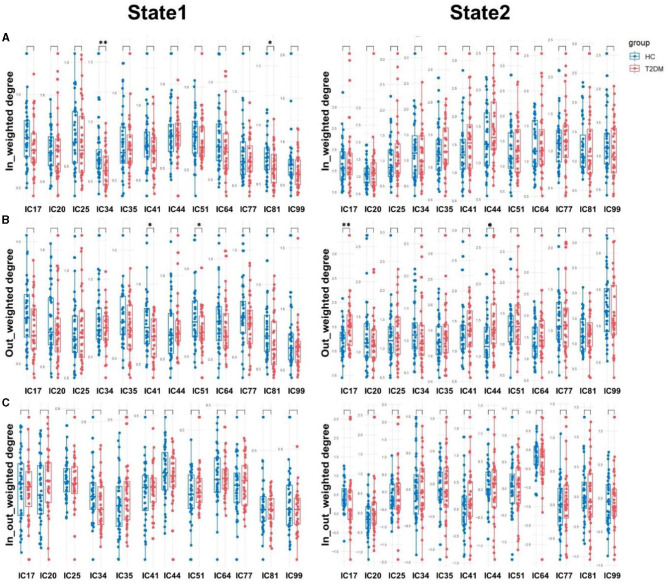
Causal influence flows of dynamic effective connectivity. The in-weighted degree **(A)**, out-weighted degree **(B)**, and in-out weighted degree **(C)** in two states for T2DM patients (red) and HCs (blue). Horizontal solid and dashed lines indicate group means and interquartile range respectively. **p < 0.01 and *p < 0.05 with FDR corrected.

**Figure 5:**
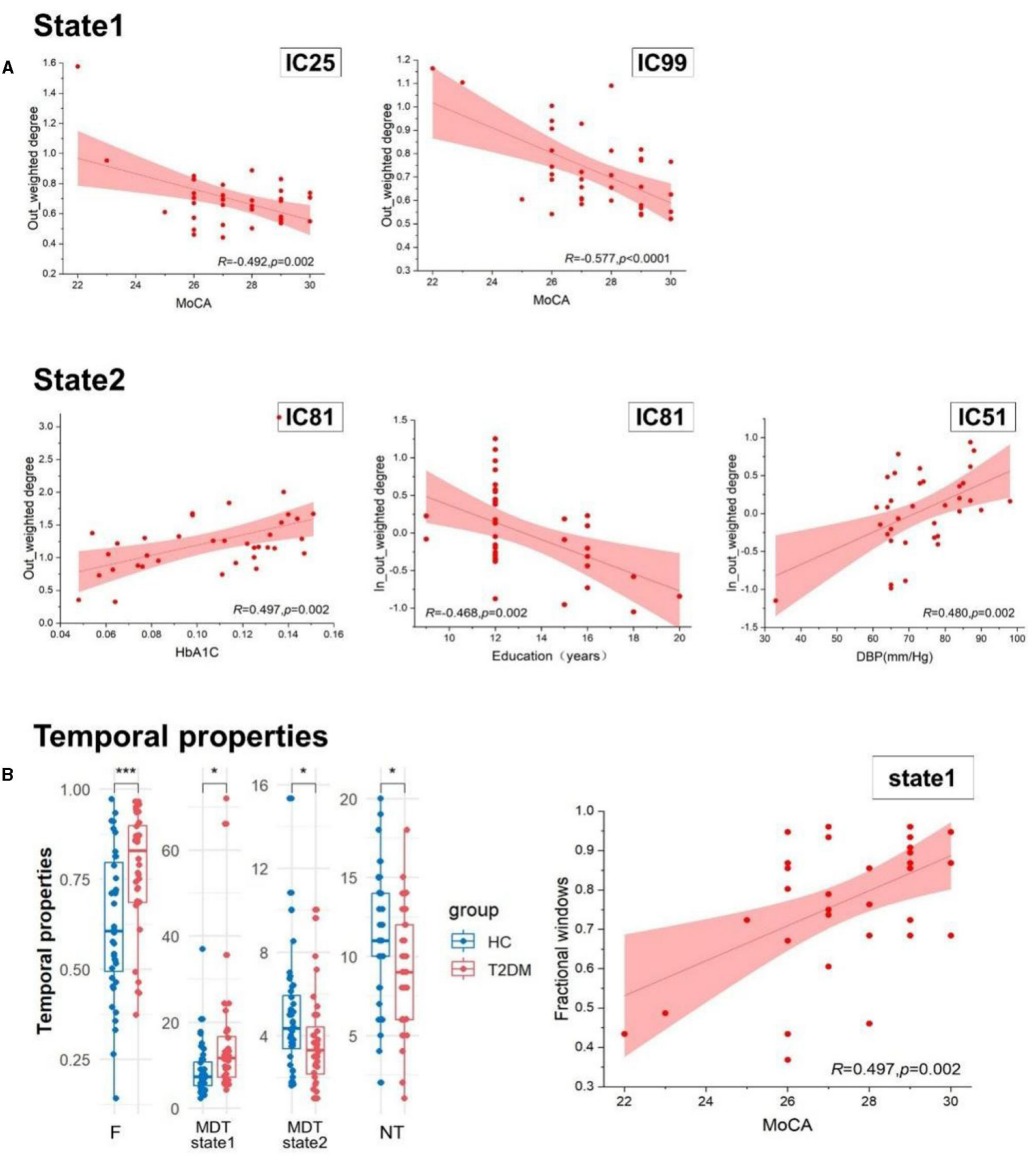
**(A)** The relationship within the casual information flow and clinical and cognitive characteristics. **(B)** The difference of temporal properties between two groups and the association of temporal properties and clinical index. HbA1c, glycosylated hemoglobin type A1C; MoCA, the Montreal Cognitive Assessment; DBP, diastolic blood pressure; F, fractional windows; MDT, mean dwell time; and NT, number of transitions. ***p < 0.001 and *p < 0.05.

By comparing the causal information flow among the two groups, it conveyed significant distinctions. In state 1, T2DM patients demonstrated reduction in the in-weighted degree of IC34 and IC81 and the out-weighted degree of IC41 and IC51. In state 2, there was no discernible difference in the in-weighted degree and in-out-weighted degree between the two groups, but the out-weighted degree of IC17 and IC44 increased in T2DM patients than HCs (*p* < 0.05). We also noticed a substantial variation in the temporal properties, with the T2DM showing larger fractional windows in state 1 and smaller in state 2, whereas the HCs showed the opposite (*p* < 0.05). As opposed to healthy subjects, the T2DM also showed an increase of mean dwell time in state 1 (*p* < 0.001), a decrease in state 2 (*p* < 0.05), and lower frequency of transitions (*p* < 0.05). Details in Figures 4, 5.

### 3.5 Relationship with clinical disease severity

The casual information flow of T2DM patients reflected downward in state 1 than healthy controls, but there was no significant difference between them in state 2. By exploring the relationships between the casual information flow and temporal properties and clinical indicators, we found that MoCA scores are negatively correlated with the out-weighted degree of IC99 (*r* = −0.492, *p* = 0.002) and IC25 (*r* = −0.577, *p* < 0.0001) in state 1. In state 2, we discovered that there is a positive correlation between diastolic blood pressure and the in-out-weighted of IC51 (*r* = 0.480, *p* = 0.002), and that HbA1C is significantly positively correlated with the out-weighted degree of IC81 (*r* = 0.497, *p* = 0.002). The education years also have a negative relationship with the in-out-weighted degree of IC81 (*r* = −0.468, *p* = 0.002). In addition, MoCA scores showed a significant correlation with fractional windows, and this correlation revealed a divergent tendency between the two states (*r* = ±0.497, *p* = 0.002). Details show in Figure 5 and Table 3.

**Table 3:** The differences of temporal properties between two groups.

**Group**	**Fractional windows**		**Mean dwell time**		**Number of transitions**
	**State1**	**State2**	**State1**	**State2**	
T2DM patients	0.77 ± 0.17	0.23 ± 0.17	15.47 ± 14.77	3.67 ± 2.24	9.03 ± 4.05
Healthy controls	0.63 ± 0.20	0.38 ± 0.20	8.86 ± 6.31	4.84 ± 2.76	11.35 ± 3.57
*P-*value	< 0.01^**^	< 0.01^**^	0.012^*^	0.047^*^	0.014^*^

^*^p < 0.05 and ^**^p < 0.01.

## 4 Discussions

In the current study, we classified the effective connectivity patterns of DMN regions into two states (the state1 shows higher frequency but lower connectivity, and the state2 shows lower frequency but higher connectivity), and observed that T2DM patients represent decreased directional effective connectivity from bilateral medial prefrontal cortex (mPFC) to middle cingulate cortex (MCC) and left inferior parietal lobule (LIPL) in state1, otherwise, these alterations are related to the impaired cognitive function in T2DM patients. To our knowledge, this study is the first to examine the effective connectivity within DMN of the group with T2DM.

The mPFC is a critical component of the default mode network, interconnecting with numerous brain areas and involved in a number of crucial processes, such as motivation, emotional control, and social behavior. As a result, it serves as a key node in the neural circuitry that mediates a variety of neurological and psychiatric disorders (30, 31). In humans, the LIPL is one of important nodes of DMN and contribute to many brain functions, especially in terms of language processing and recognition memory. Furthermore, recent neuroimaging studies have revealed that the existence of additional functions of LIPL, including attention, action and salience processing (32–36). Available literatures found that bilateral mPFC and LIPL jointly participant in the functions of emotional regulation, memory and executive function (37–39). Based on this discovery, it is reasonable to speculate that the decreased connectivity from mPFC to LIPL may be related to the injury of emotion, memory and executive function in T2DM patients and ultimately leading to the onset of cognitive disorders, but further investigation will be required. Furthermore, we discovered that the out-weighted degree of LIPL decreases in state 1 as well as the in-out-weighted degree of LIPL is positively correlated with DBP in state 2. Combining the above results, we can observe that the abnormality of causal influence flows of LIPL is more prominent in state 1, however, in the correlation analysis, the abnormality is mainly reflected in state 2, which may be due to the fact that the abnormality is more prominent in state 1 because of the lower level of activity in state1, whereas state 2 is more likely to be affected by the clinical factors due to the higher activity. In conclusion, we can speculate that T2DM patients have greater damage in the LIPL node, and that this damage may be linked to abnormal blood pressure. Additionally, although the difference between T2DM patients' DBP and that of healthy individuals was not statistically significant, but T2DM subjects' DBP was slightly lower indeed. A number of studies have examined the relationship between blood pressure and cognitive functioning in T2DM and have yielded significant results (40–42), therefore, future research is necessary to determine the precise mechanisms within T2DM patients' brain activity and blood pressure.

The middle cingulate cortex (MCC) is a hub for dynamic switching between emotion and cognition, where motions with potentially punitive implications, such as suffering and menace, can be integrated into regulatory areas to express dread and agitation, facilitating goal-directed behaviors, and inclining the focus of selective attention. Neuroimaging findings suggested that the neural circuit of the MCC/mPFC was significantly activated by pains and then regulated the expression of dread and agitation (43). The decreased DEC from mPFC to MCC in T2DM potentially be associated with nerve demyelination and peripheral neuropathy and lead to the reduced response to pain and the impaired cognition, yet this connection remains to be explored. Additionally, we discovered a negative relationship between MoCA scores and the out-weighted degree of MCC in state 1. It may be inferred that as the out-weighted degree of MCC increase, there is a subsequent decrease in cognitive function of T2DM, which could potentially be accompanied by a drop in the in-weighted degree. This would exacerbate the impairment of MCC. Given the two findings, it becomes sense to speculate that cognitive dysfunction and MCC node damage are intimately associated in T2DM patients.

According to previous research findings, the causal information flows among the DMN regions were correlated with their neuronal activity levels (44). The causal information flows of diabetic patients revealed a trend of decreasing in state1 and increasing in state2, suggesting that neuronal activity turned to be weakened in state1 and enhanced in state2 in T2DM compared with healthy controls. Although there was no difference in the cognitive function of the two groups, this change may be the potential mechanism of early cognitive decline of T2DM. During the analysis of causal information flows, we found that the in-weighted degree of bilateral fusiform gyrus (FFG, IC34) and middle frontal gyrus (MFG, IC81) and the out-weighted degree of bilateral precuneus (PCU, IC41) and LIPL (IC51) all declined in state 1, meanwhile, the out-weighted degree of bilateral posterior cingulate cortex (PCC, IC17) and right angular gyrus (Ang, IC44) increased in state 2, which possibly represented that the dynamic activity of these areas may be most susceptible to be damaged within DMN regions in patients with T2DM.

As the brain region with the strongest metabolic activity and functional connectivity, the PCC has been argued to be the core hub of DMN. Previous T2DM studies have found hypoconnectivity reaching peak in the PCC, relating with the cognitive impairment of recall memory, transient memory executive function (5, 45). The fusiform gyrus (FFG) is crucial for advanced-level object recognition and is associated with various neural pathways involved in processing visual food cues. The middle frontal gyrus (MFG) is a part of the dorsolateral prefrontal cortex and plays a key role in dietary control, food craving, and metabolic regulation (18, 46). The PCU is also a core node of the DMN, exhibiting a remarkably high metabolic rate and serving as a linchpin in facilitating the synthesis of external information and internal representations (like episodic memories, self-relevant information, and subjective value processed by other DMN regions) (47). The right Ang has been shown to be a highly consistent cluster that is most likely to be involved in attention reorienting processes, semantic processes and memory (48). To summarize, the majority of the previously mentioned brain regions are intimately linked to metabolic functions. Therefore, our research findings are consistent with the previously mentioned results, diabetes as a metabolic disease that impacts these regions' neural activity and ultimately resulting in altered cognitive function. Furthermore, we found a strong association between HbA1C and education level with the out-weighted degree and in-out-weighted of MFG in state 2 in our correlation analysis. As HbA1C increased, the out-weighted degree of MFG also increased; however, as the education level increased, the in-out-weighted of MFG decreased, illustrating that impaired blood glucose will affect the function of MFG and that this effect will be more severe as the level of education decreases. Significantly, this effect is more prominent in state 2 with higher levels of brain activity than state 1, which may strengthen the conclusion that patients with diabetes may have reduced cognitive performance due to dysregulation of glucose metabolism. It might be conjectured that this impact is particularly severe in MFG.

Moreover, we found that the out-weighted degree of bilateral hippocampus (Hip, IC25) in state1 is negatively correlated with MoCA scores of T2DM. Early cognitive decline of diabetes was mainly manifested in memory, as we all know that the key responsibility of the hippocampus, therefore, our study further confirmed that the reduced activity of brain regions will correspondingly influence cognitive functions of T2DM. In addition, from the perspective of temporal properties, we can saw that T2DM patients stay in state1 longer than in state 2 compared to HCs, and show a decreased rate of transitions between two states, which indicates the lower level of brain activity in diabetic patients. And by calculating the relationship between temporal properties and clinical features, the fractional windows showed a negative correlation with MoCA scores in state2 and the opposite in state1, which further conveys that lower activity of our brain may be the early manifestations of cognitive impairments in diabetes. Existing research has identified closely relationships between abnormalities in the temporal properties of DEC and cognitive impairment (49, 50). In summary, there is a strong association between abnormal glucose metabolism, reduced brain activity and cognitive impairment in patients with T2DM.

## 5 Limitation

There are still some restrictions on our investigation. First, the sample size of our study still remained too small with a total of 36 T2DMs and 40 HCs were included. As a common disease, diabetes was generally studied in a large scale of subjects. Second, the EPI sequence in our investigation scanned 180 volumes within 6 min, however, more sophisticated sequences, like the multi echo technique, can scan more volumes in a shorter time. Third, the current study was still a cross-sectional study, and in the future longitudinal studies should be designed to explore the temporal variation of DEC in T2DM patients.

## 6 Conclusion

By using the techniques of ICA and mGCA, our study discovered that patients with T2DM show decreased DEC, lower brain activity and abnormalities of causal information flows within DMN regions compared to HCs. Additionally, there is a strong correlation between low brain activity status and clinical characteristics and cognitive impairment in diabetes, which may serve as early image markers for further predicting cognitive decline in this patient population. Notably, there were crossovers of these results in some brain regions within DMN which are most severely impaired in T2DM with early cognitive decline in our hypothesis and need to be further explored in the future.

## Data availability statement

The raw data supporting the conclusions of this article will be made available by the authors, without undue reservation.

## Ethics statement

The studies involving humans were approved by the Ethics Committee of Lanzhou University Second Hospital (no. 2022A-377). The studies were conducted in accordance with the local legislation and institutional requirements. The participants provided their written informed consent to participate in this study.

## Author contributions

KX: Investigation, Methodology, Writing—original draft. JW: Conceptualization, Data curation, Methodology, Software, Writing—review & editing. GL: Project administration, Writing—review & editing. JY: Formal analysis, Writing—review & editing. MC: Formal analysis, Writing—review & editing. LJ: Formal analysis, Writing—review & editing. JZ: Funding acquisition, Project administration, Supervision, Writing—review & editing.
